# Surgical Approach to a Post-traumatic Fat Fracture

**DOI:** 10.7759/cureus.3378

**Published:** 2018-09-27

**Authors:** Joseph El Khoury, Diane Adams, Christian Saliba, Johnny El Rayes, Audrey Massin, Claire Fenoll

**Affiliations:** 1 Plastic Surgery, Hospital Saint Joseph De Paris, Paris, FRA; 2 General Surgery, Lebanese American University-Medical Center, Beirut, LBN; 3 Orthopedics, Saint Joseph University, Beirut, LBN; 4 Radiology, Centre Chirurgical Des Princes, Paris, FRA

**Keywords:** fat fracture, pseudolipoma, lipofilling, fat transfer

## Abstract

This paper describes the surgical findings and operative approach to treating a post-traumatic pseudolipoma. To the best of our knowledge this is the first case report that details the surgical technique of removal of a pseudolipoma and aesthetic restitution of the area. We used a combined enucleation of the mass, an extensive rigotomy and fat transfer in order to achieve adequate result.

## Introduction

Post-traumatic pseudolipoma (PPL), also known as fat fracture, is caused by acute, chronic or repetitive trauma. It consists of a benign tumor with an unclear pathophysiology. Some believe that disruption of the fatty layers and inflammatory mediators could be responsible its formation [1]. Others postulate that the fibrous capsular formation is due to vascular insufficiency [2]. It is crucial to make the differentiation between this benign tumor and a liposarcoma or any other malignant processes [3]. The magnetic resonance imaging (MRI) and sonography seem to show a cleavage plane between the PPL and the tissue around it [4]. The usual treatment consists of excision of the PPL in totality with its bursa or capsule and redistribution of the fatty layers to avoid depression [5]. Here we present the case of a patient whom the usual treatment did not offer the optimal aesthetic result. Therefore we opted for an additional procedure during the same operation, to achieve a complete satisfactory result.

## Case presentation

This is a case of a 53-year-old female patient who presented to our hospital for a cosmetic deformity. She suffered from a direct trauma to her lateral right thigh two years ago. She had no recall of a hematoma formation. She had no continuous pain but feels traction on hip abduction. Her main complaint is the difficulty to wear tight clothes.

**Figure 1:**
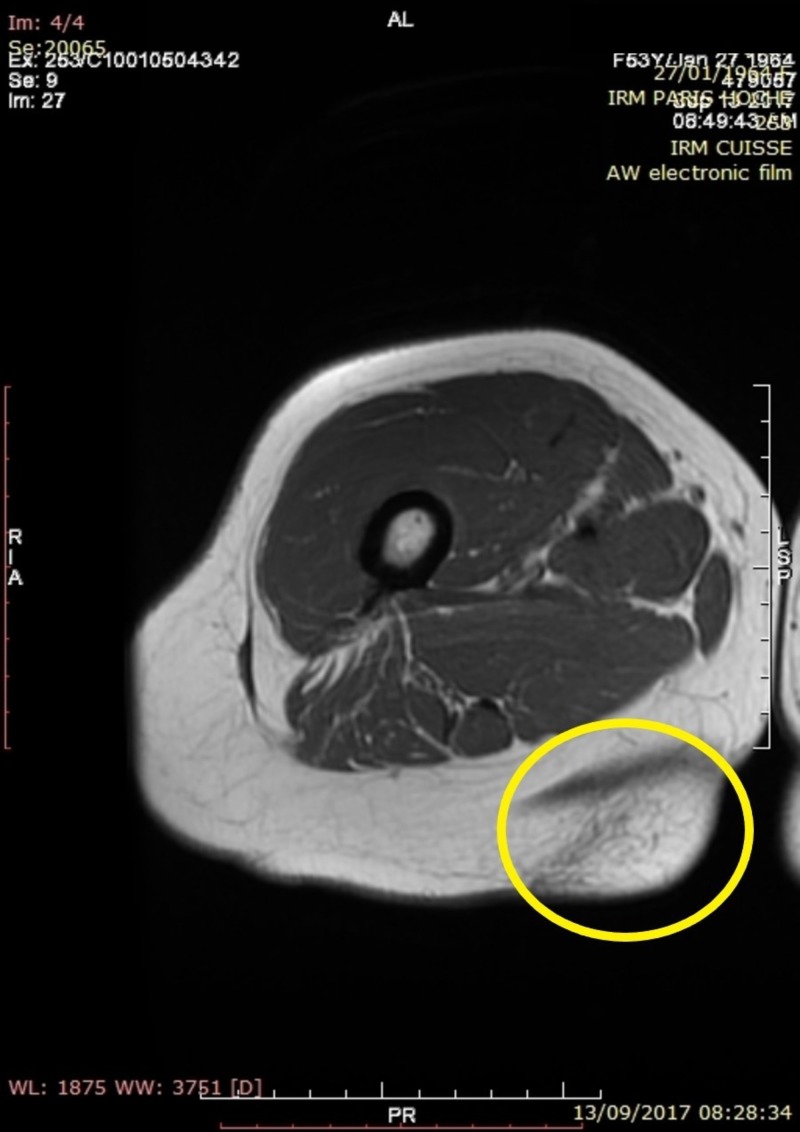
Axial view of a lower extremity MRI showing the pseudolipoma marked by the yellow circle. MRI: Magnetic resonance imaging

**Figure 2:**
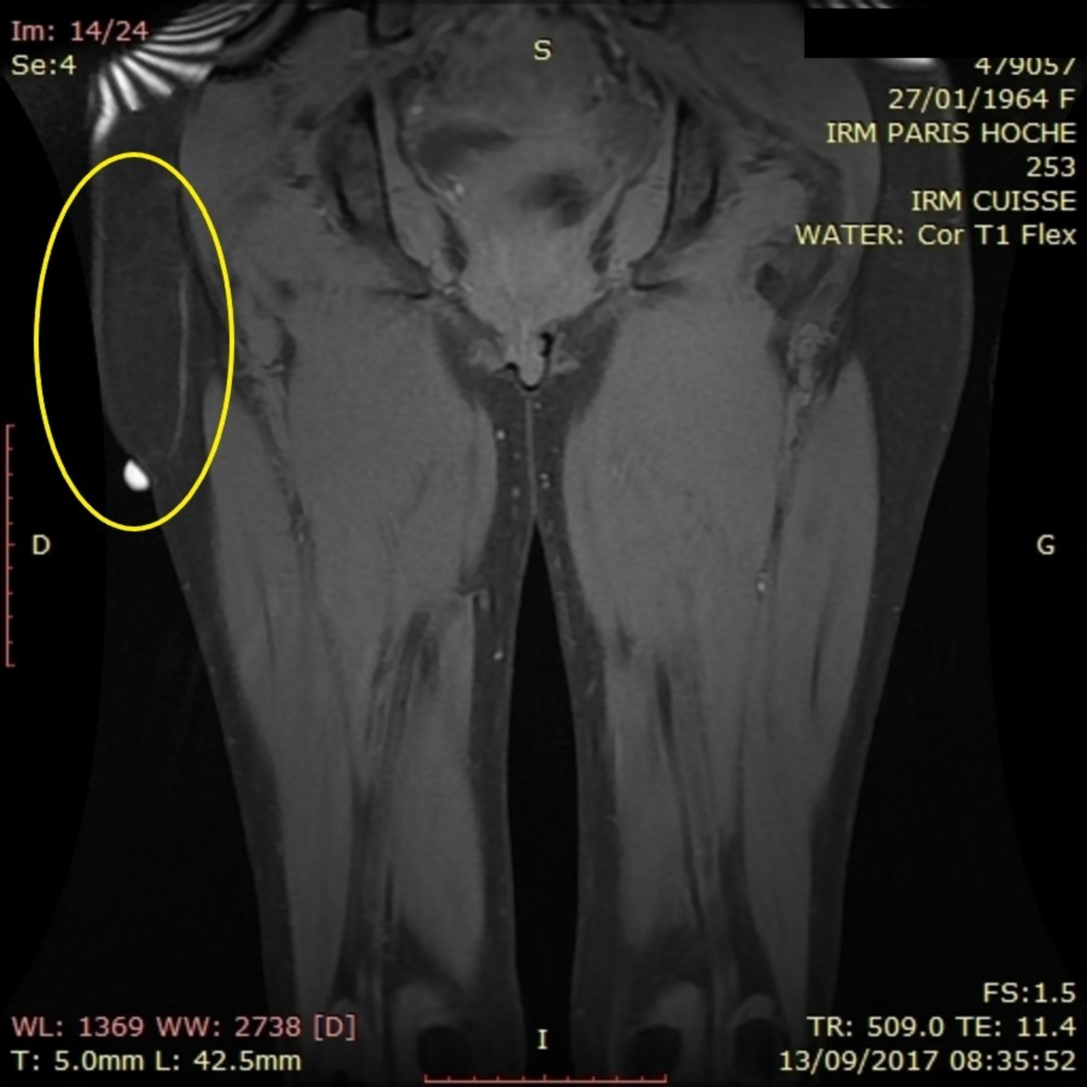
Coronal view of a lower extremity MRI showing the pseudolipoma in the yellow circle. MRI: Magnetic resonance imaging

A lower extremities MRI was performed showing a 5 x 7 x 2 cm cystic cavity that created a gliding plane between the subcutaneous tissue and the underlying muscular fascia (Figures 1, 2). With the patient history in mind the diagnosis of pseudolipoma was postulated.

**Figure 3:**
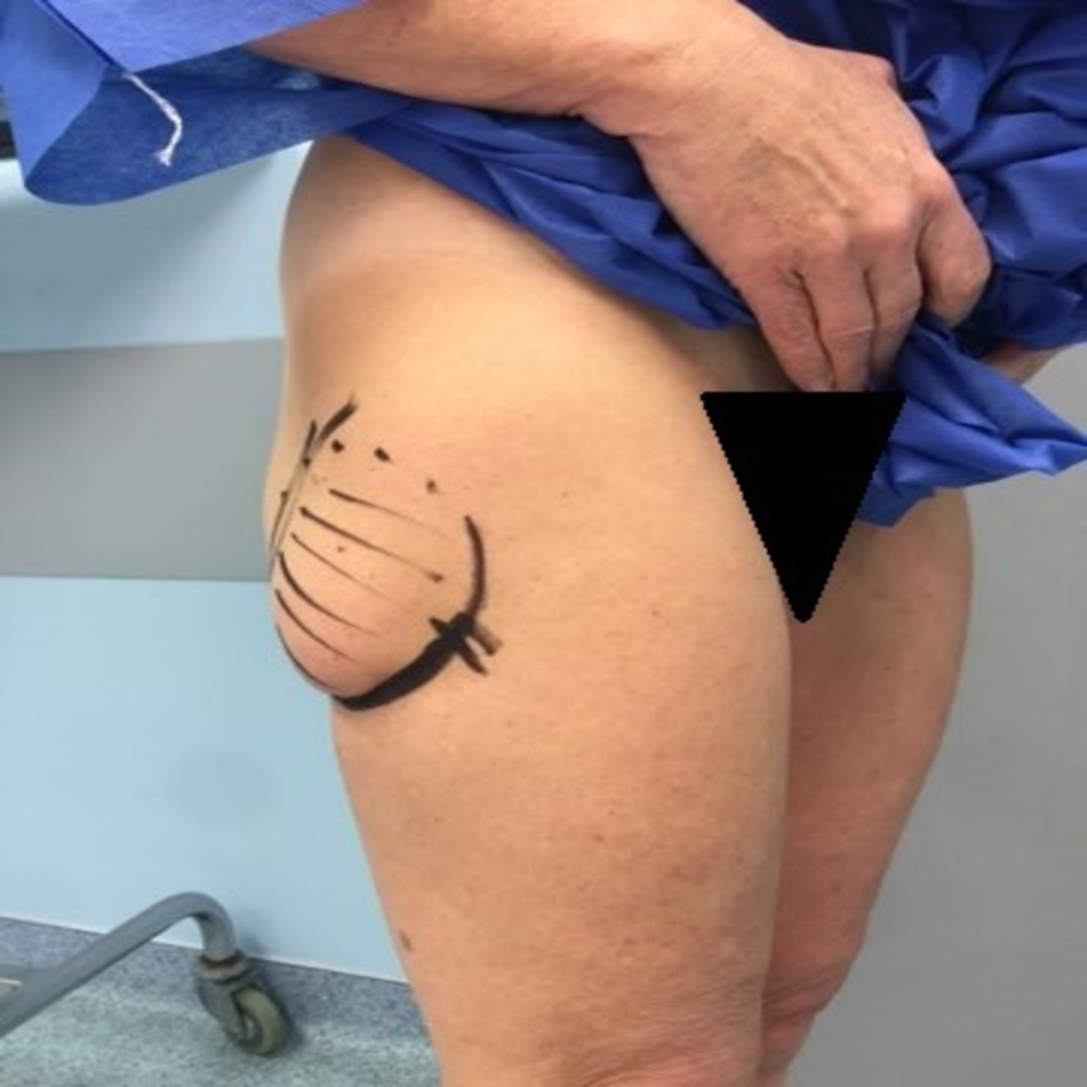
Surgical marking of the area of interest.

The pseudolipoma is marked preoperatively (Figure 3) and a 5 cm incision on the caudal end of the mass is highlighted. We chose this incision in order to access this fibrotic adherence that caused the stair step deformation.

**Figure 4:**
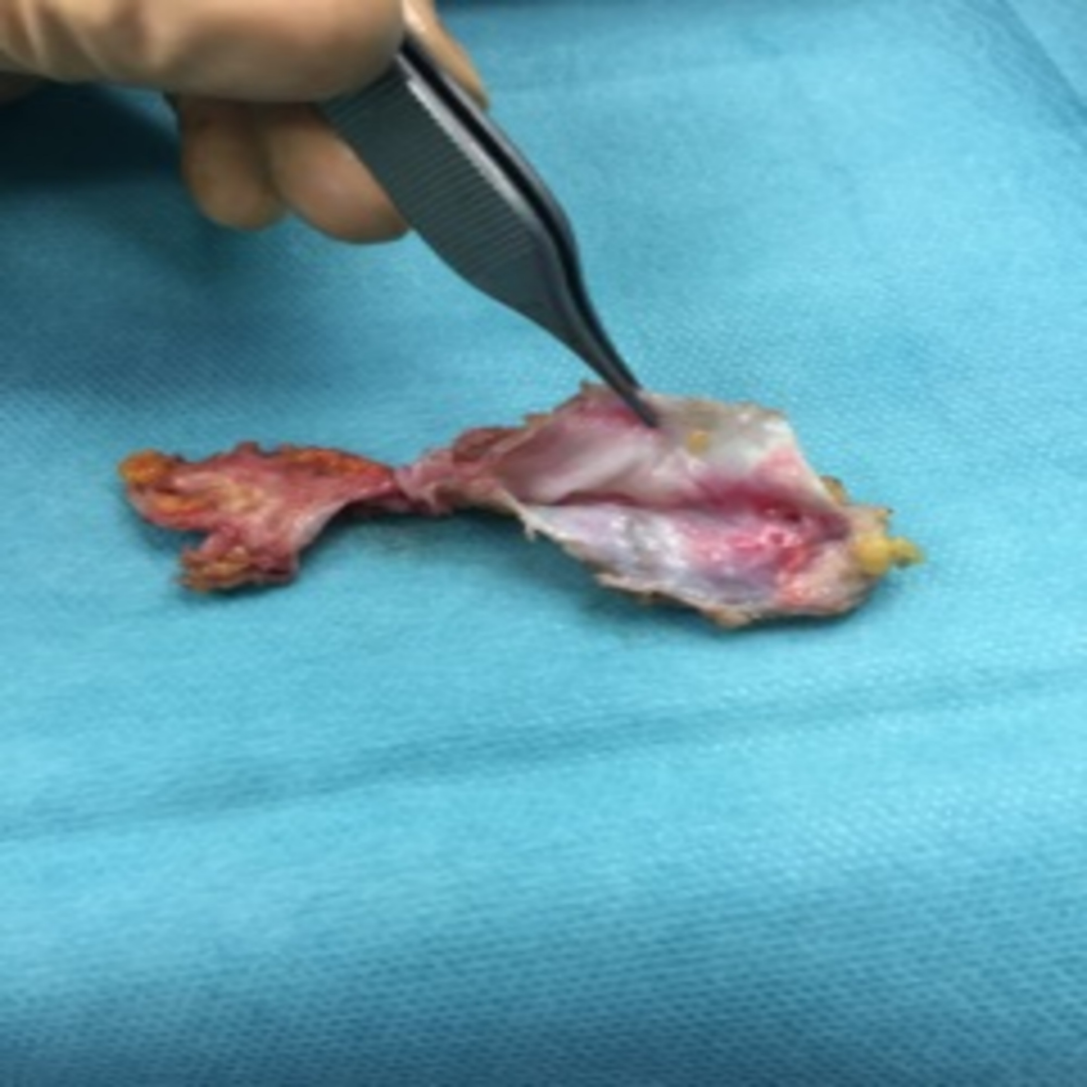
Pseudolipoma with its capsula.

The operation was performed under general anesthesia. The surgical field included the whole leg. The incision was carried to the subcutaneous tissue and blunt dissection was performed to the underlying fascia. A finger dissection was performed between the muscular fascia and the posterior capsule of the pseudolipoma and a cautery dissection on the anterior surface of the mass. After extraction, the tumor consisted of a serous filled capsular formation (Figure 4).

**Figure 5:**
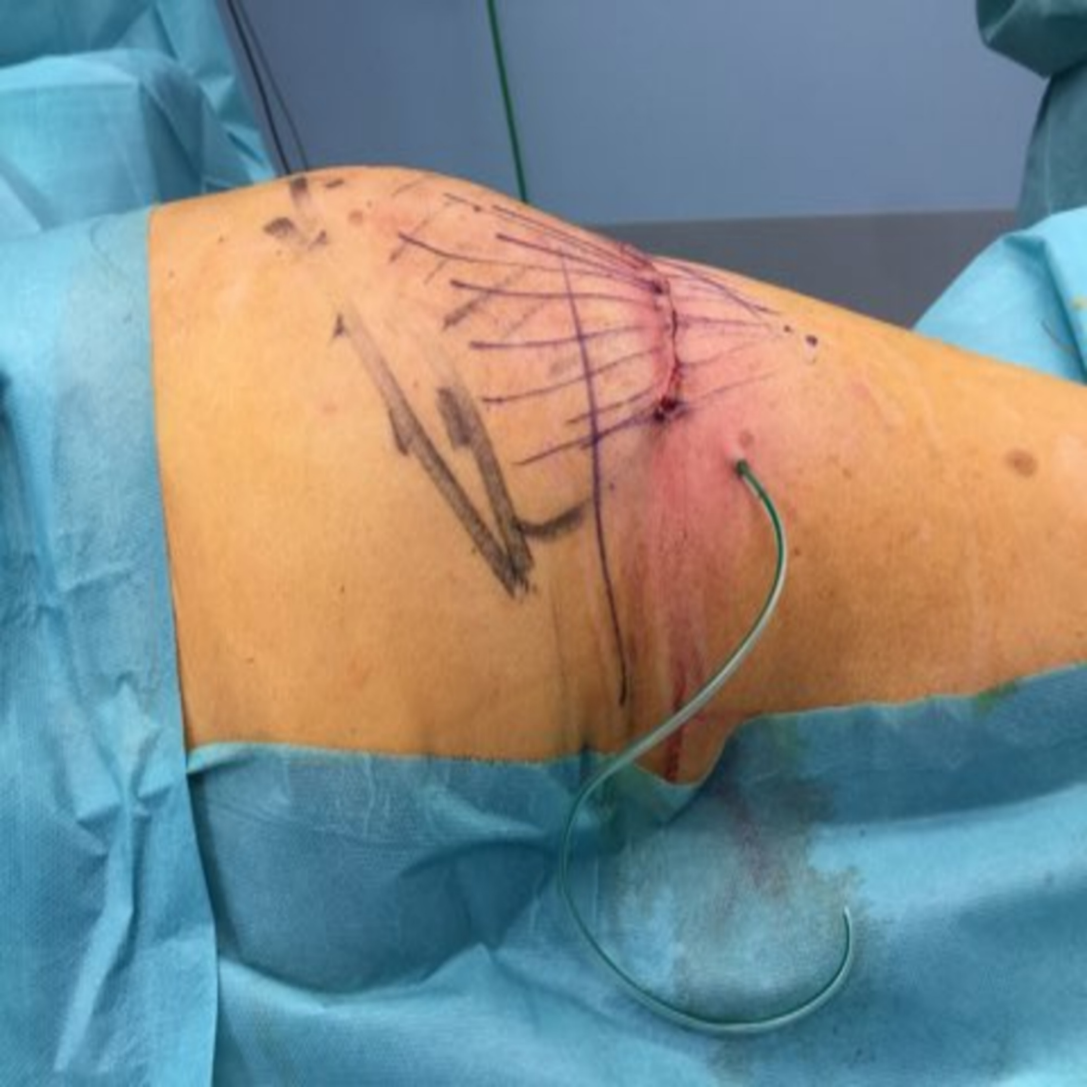
Immediate post-operative result prior to the fat transfer procedure.

After removal and wound suture we can notice a depression in the area (Figure 5).

An extensive rigotomy using a Coleman three cannula was performed to remove any remaining adherences.

**Figure 6:**
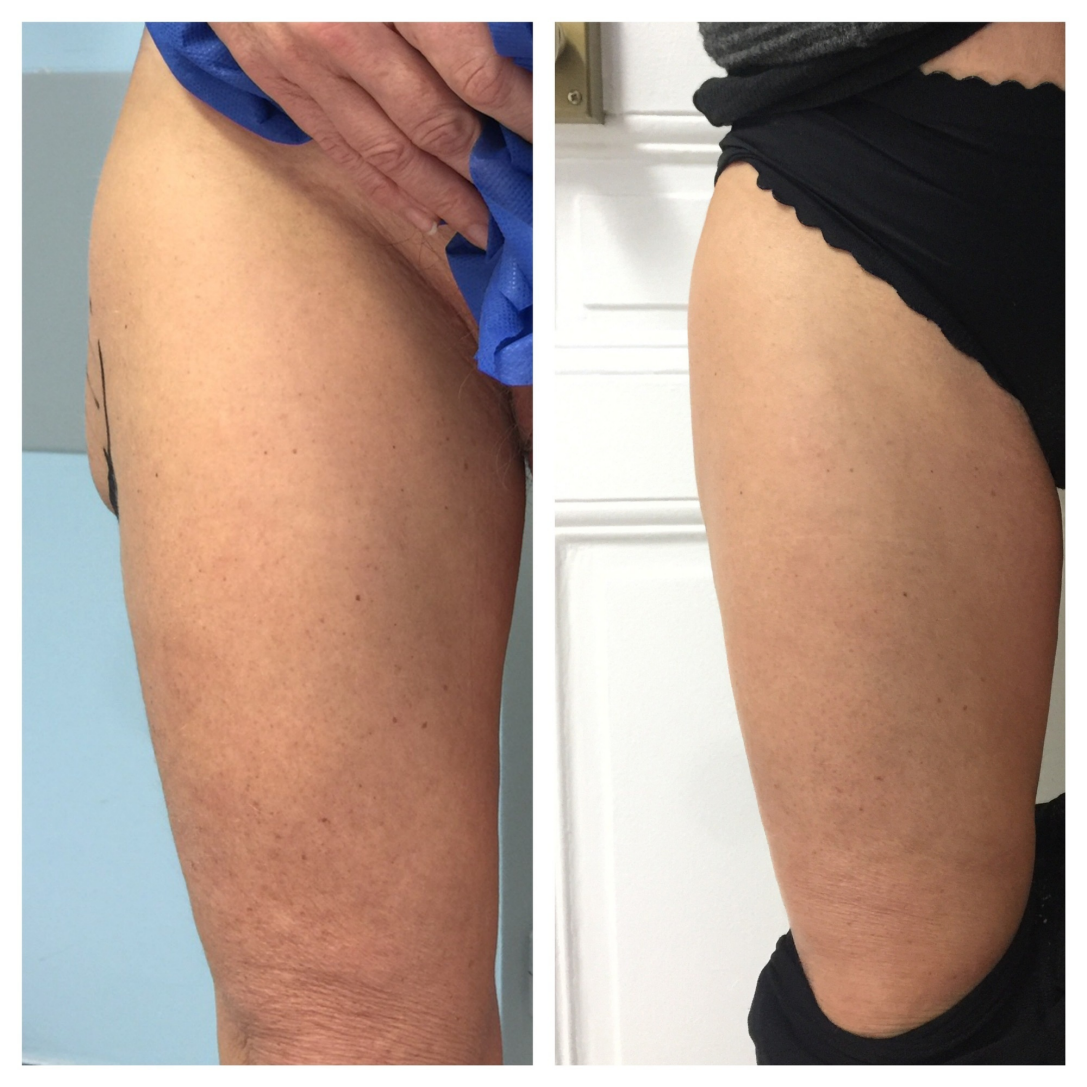
Comparison between pre-operation and three months post-operation. The image on the left was taken directly pre-operation and shows a clinically apparent hump. The image on the right was taken three months post-operation and shows the aesthetic results with a regular contour without any hump or skin depression.

A total of 20 cc of fat were extracted from the inner thigh using a tumescent solution with a Coleman three cannula and reinjected by a fanning technique to achieve regular contour. Aesthetic results three months following the operation were encouraging and showed a regular contour without any hump sign or skin depression (Figure 6).

## Discussion

In this case, after removal of the mass we noticed that fat redistribution was not sufficient to achieve the optimal aesthetic result. We then opted to perform a lipofilling of the area using the Coleman technique. 20 cc of purified fat were transferred from the medial thigh to reshape the contour creating a smoother transition.

In the last five years the senior author has encountered multiple cases of fat fractures treated with the same manner. It is important to note that only performing liposuction and/or rigotomy are in no use in similar case. The fact that fat trauma causes a capsular formation is indicative that the logical solution is performing surgical capsulectomy. The plane of dissection can be sometimes hard to follow in some cases while in others only finger dissection is sufficient. Finally, with the advances in fat transfer techniques and indications, immediate defect reconstruction can be achieved in a single stage.

## Conclusions

The usual treatment of PPL consisting of its excision with its capsule and redistribution of the fatty layers is not always sufficient to avoid skin depression. To achieve optimal aesthetic results, we advocate to perform a lipofilling of the area using the Coleman technique. This is now achievable in a single stage procedure due to advances in fast transfer techniques.
